# Efficient monitoring of the blood-stage infection in a malaria rodent model by the rotating-crystal magneto-optical method

**DOI:** 10.1038/srep23218

**Published:** 2016-03-17

**Authors:** Ágnes Orbán, Maria Rebelo, Petra Molnár, Inês S. Albuquerque, Adam Butykai, István Kézsmárki

**Affiliations:** 1 Department of Physics, Budapest University of Technology and Economics and MTA-BME Lendület Magneto-optical Spectroscopy Research Group, 1111 Budapest, Hungary; 2 Instituto de Medicina Molecular, Faculdade de Medicina Universidade de Lisboa, 1649-028 Lisbon, Portugal

## Abstract

Intense research efforts have been focused on the improvement of the efficiency and sensitivity of malaria diagnostics, especially in resource-limited settings for the detection of asymptomatic infections. Our recently developed magneto-optical (MO) method allows the accurate quantification of malaria pigment crystals (hemozoin) in blood by their magnetically induced rotation. First evaluations of the method using β-hematin crystals and *in vitro P. falciparum* cultures implied its potential for high-sensitivity malaria diagnosis. To further investigate this potential, here we study the performance of the method in monitoring the *in vivo* onset and progression of the blood-stage infection in a rodent malaria model. Our results show that the MO method can detect the first generation of intraerythrocytic *P. berghei* parasites 66–76 hours after sporozoite injection, demonstrating similar sensitivity to Giesma-stained light microscopy and exceeding that of flow cytometric techniques. Magneto-optical measurements performed during and after the treatment of *P. berghei* infections revealed that both the follow up under treatment and the detection of later reinfections are feasible with this new technique. The present study demonstrates that the MO method – besides being label and reagent-free, automated and rapid – has a high *in vivo* sensitivity and is ready for in-field evaluation.

The study of human malaria, one of the most widespread infectious diseases of the globe, is an important agenda of today’s scientific research. It involves various methods ranging from epidemiological analysis and clinical studies to laboratory model systems such as *in vitro* parasite cultures in human red blood cells (RBCs) and *in vivo* rodent models. Rodent models of malaria have been widely used to study the biology and pathology of malaria (reviewed in ref. 1) as well as for *in vivo* evaluation of novel vaccine and drug candidates^2^,^3^. Rodent models were also applied to successfully investigate the host immune response (reviewed in ref. 1) despite their limitations to replicate certain aspects of the human disease^4^,^5^.

Under natural conditions the *Plasmodium* life cycle in the mammalian host starts with the inoculation of sporozoites into the skin by parasite-infected mosquitos. After inoculation the parasites rapidly reach the liver where they infect hepatocytes and develop into thousands of exoerythrocytic merozoites. Once released from the hepatocyte, the merozoites infect red blood cells, leading to the blood stage of the infection that is characterized by cyclic asexual replication. The duration of the liver development and the length of the asexual cycle varies between species. In the case of the *P. berghei* rodent parasites investigated here, the asymptomatic liver stage lasts 47–52 hours and the length of one erythrocytic cycle is approx. 24 hours^6^,^7^. Experimental challenges can be carried out either via the bites of infected mosquitos, or through the artificial injection of liver-infecting sporozoites or infected erythrocytes. The progression of the blood stage is quantitatively characterized by the percentage of infected RBCs, i.e. the parasitemia.

The reference method of diagnostics, light microscopy of Giemsa-stained thin blood smears, is a labour-intensive procedure relying on the expertise of the investigator. On the other hand, more sensitive molecular methods based on polymerase chain reaction (PCR) are still not used for either the continuous monitoring of laboratory experiments or for the primary diagnosis of patient samples due to their high running cost^8^,^9^. In laboratory studies automated approaches, such as flow cytometry are preferred and extensively used. However, to achieve sufficient sensitivity they often require special dyes combined with complex protocols^10^,^11^,^12^ or transgenic fluorescent protein-expressing parasites^13^,^14^,^15^. The use of transgenic luciferase expressing parasites has also been shown to accurately evaluate the pre-patent period, i.e. the time between sporozoite inoculation and the appearance of parasites in the peripheral circulation, and the early blood stages in a novel bioluminescent assay^16^. However, methods exploiting the chemiluminescent properties, besides being costly, are limited to animal models.

The need for a universally applicable and automated method for diagnostics has motivated extensive research on malaria pigment (hemozoin), a natural biomarker of the infection. In particular, various magnetic and/or optical detection schemes targeting hemozoin have been proposed^17^,^18^,^19^,^20^,^21^.

Hemozoin is a micro-crystalline heme compound produced by all *Plasmodium* spp. during the intraerythrocytic stage as they detoxify free heme derived from hemoglobin digestion^22^,^23^,^24^. The steadily increasing hemozoin content of the parasites acts as an optimal indicator of their maturation during the erythrocytic cycle. Indeed, the hemozoin-based flow cytometric detection of parasite maturation has been utilized in a novel reagent-free drug sensitivity assay as well^25^,^26^.

The rotating-crystal magneto-optical (MO) technique determines the concentration of hemozoin crystals via their linear dichroism and magnetic anisotropy^27^. In our recent study the sensitivity of the method has been evaluated using ring and schizont stages from *P. falciparum in vitro* cultures where detection thresholds of 0.0008% and 0.0002% parasitemia have been found, respectively^28^. These results implied that the MO method is suitable for the high-sensitivity detection of the infection as a laboratory tool or, eventually, as an in-field diagnostic technique.

Here we test the MO method *in vivo* by monitoring the onset of the blood-stage after sporozoite challenge in a malaria mouse model using *P. berghei* parasites. To evaluate the sensitivity of the MO technique light microscopy, PCR and flow cytometry were used as control methods. The clearance of hemozoin during the treatment of *P.* berghei infections was also followed to establish a time-frame after which the MO signal vanishes in successfully treated mice.

## Results

### Onset of the blood-stage in *P. berghei* infections monitored by the MO method, light microscopy, PCR and flow cytometry

The onset of the blood-stage following *P. berghei* sporozoite challenges was monitored in three independent experiment series (A, B and C). Mice in series A and B were infected intravenously, while mice in series C were subjected to mosquito bites (for details see Methods). In order to evaluate the sensitivity of the MO method in comparison with standard techniques and to determine the time scale of the first positive detections, blood was drawn from mice starting at the end of the liver stage in approx. 5-hour intervals until the fourth day of blood-stage infection. The blood samples were examined to assess the appearance of the first parasites by thin smear microscopy, flow cytometry and MO measurements i) from 48 h to 132 h post infection (pi) in series A, ii) from 70 h to 132 h pi in series B and iii) from 56 h to 166 h in series C. For the last two methods the detection limit was determined as the average value plus three times the standard deviation of the data measured for the uninfected references (for details see Methods). Additionally, q-PCR measurements were performed in the early time points of the intraerythrocytic parasite development for series A and B.

**Figure 1:**
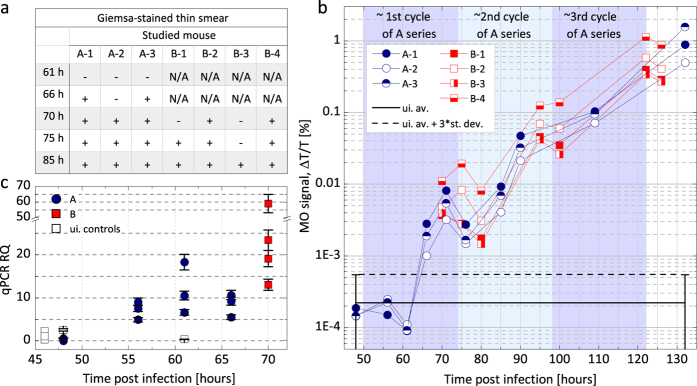
Onset of the blood-stage in series A and B (intravenously infected mice) monitored by light microscopy, the magneto-optical method and q-PCR. (**a**) Microscopic examination of Giemsa-stained thin blood smears of mice in series A and B. + signals indicate that at least one parasite was found in the whole of the smears (approx. 20–40 fields), − signals mean that no parasites were detected in the smears. (**b**) The results of the MO measurements. Each circle (square) represents the MO signal of an infected mouse in series A (B) at a given time point after sporozoite injection. The continuous black line represents the average of the MO values of five uninfected control mice measured in all time points. The dashed black line is the detection limit defined as the average plus three times the standard deviation of the uninfected values (for details see Methods section). The MO signals of series A exceed the detection limit at 66 h pi. The MO signals of all mice in series B exceed the detection limit already at the first, 70 h sampling point. The background shading illustrates the estimated layout of the first three erythrocytic cycles of series A. (**c**) Results of the q-PCR measurements plotted by circles (squares) for series A (B). The error bars represent the standard deviation of technical duplicates. The □ represent measurements on three uninfected controls and the real-time control mouse at 48 h and 61 h.

**Figure 2:**
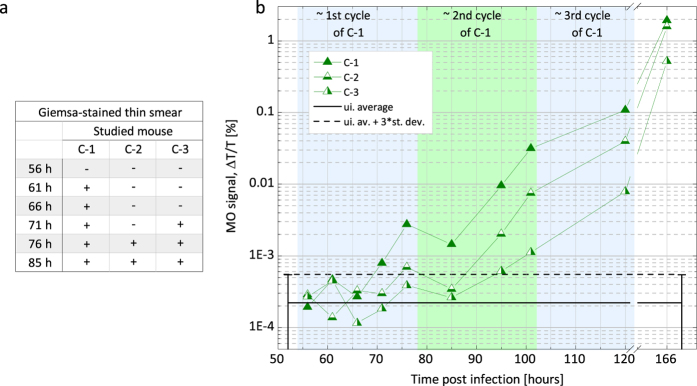
Onset of blood stage infection of series C (mosquito-infected mice) monitored by light microscopy and the magneto-optical method. (**a**) Microscopic examination of Giemsa-stained thin blood smears of mice in series C. + signals indicate that at least one parasite was found in the whole of the smears (approx. 20–40 fields), while − signals mean that no parasites were detected in the smears. (**b**) The results of the MO measurements. Each triangle represents the MO signal for a given mouse in series C at a given time point after the mosquito challenge. The continuous black line represents the average of the MO values of five uninfected control mice measured in all time points. The dashed black line is the detection limit defined as the average plus three times the standard deviation of the uninfected values (for details see Methods section). The MO signals of mouse C-1, C-2 and C-3 exceed the detection limit at 71 h, 76 h and 95 h, respectively. The background shading illustrates the estimated layout of the first three erythrocytic cycles.

The Giemsa-stained thin blood films showed the first parasites in the circulation of mice in series A and B between 66–75 h pi with one exception, when parasites were observed only at 85 h pi (Fig. 1a). The first occurrences of positive microscopy results show small variation in these controlled infections and coincide well with the estimated end of the first erythrocytic cycle (approx. 70–75 h). In series C the first parasites were observed in the same time interval (61–71 h pi), however in these cases only single parasites were found in the smears until 76 h pi (Fig. 2a).

Real-time PCR analysis was performed on blood samples drawn from mice in series A between 48–66 h pi and from mice in series B at 70 h pi. Results in Fig. 1c show that the difference in the PCR signal between the infected and non-infected samples becomes significant at 56 h pi, and gradually increases for later samplings. These PCR data are in good agreement with previous findings described by Zuzarte-Luis *et al.*^16^ under similar experimental settings and they confirm the onset of the blood stage with the expected high sensitivity.

**Figure 3:**
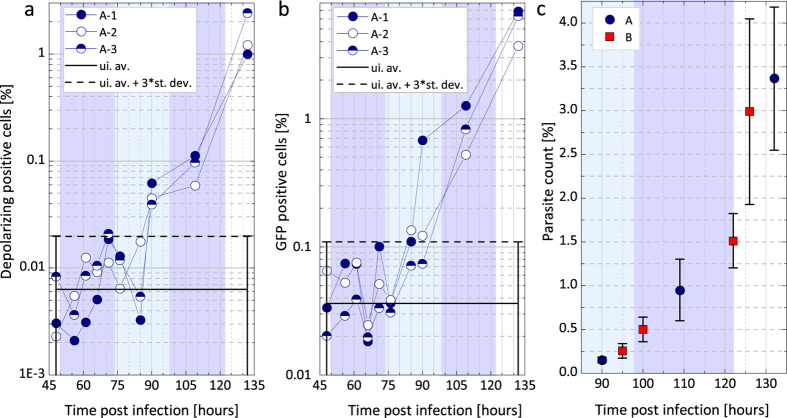
Monitoring parasitemia during the progression of the infection in series A and B via flow cytometry and light microscopy. (**a**) The results of the DSS measurements. The circles represent the percentages of DSS positive events in the total population of RBC counts for a given mouse in series A. The continuous black line represents the average signal of the uninfected reference, while the dashed black line is the detection limit defined as the average plus three times the standard deviation of the uninfected values (for details see Methods section). The DSS signals of all mice in series A unambiguously exceed the detection limit at 90 h pi. (**b**) The results of the GFP measurements. The notation of the symbols is the same as in panel (**a**). The GFP percentages of mouse A-1, A-2 and A-3 exceed the detection limit (dashed line) at 85 h, 85 h and 109 h pi, respectively. The background shading illustrates the estimated layout of the first three erythrocytic cycles. Note the different vertical scales in graphs (**a**,**b**). (**c**) Averaged parasitemia values determined by light microscopy after 90 h pi at the inspected time points of series A (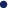) and B (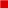).

**Figure 4:**
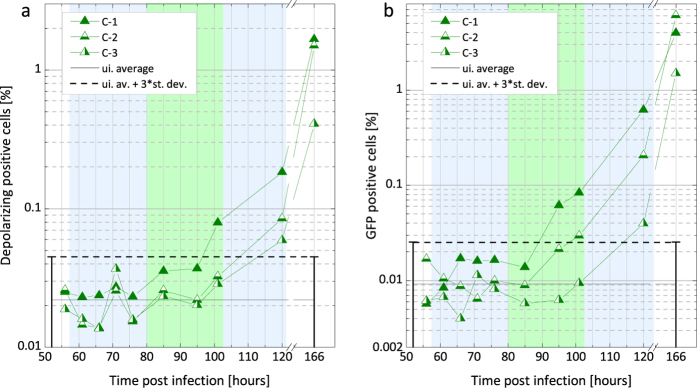
Monitoring parasitemia during the progression of the infection in series C via flow cytometry. (**a**) The results of the DSS measurements. The triangles represent the percentages of DSS positive events in the total population of RBC counts for a given mouse in series C. The continuous black line represents the average signal of the uninfected references, while the dashed black line is the detection limit defined as the average plus three times the standard deviation of the uninfected values (for details see Methods section). The DSS percentages of mouse C-1, C-2 and C-3 exceed the detection limit at 101 h, 120 h and 120 h pi, respectively. (**b**) The results of the GFP measurements. The notation of the symbols is the same as in graph (**a**). The GFP percentages of mouse C-1, C-2 and C-3 exceed the detection limit (dashed line) at 95 h, 100 h and 120 h pi, respectively. The background shading illustrates the estimated layout of the first three erythrocytic cycles. Note the different vertical scales in graphs (**a**,**b**).

Flow cytometric analysis was also performed for measurement series A and C (Figs 3 and 4, respectively). In these experiments two different parameters were assessed simultaneously: i) fluorescence of GFP expressing parasites^13^ and ii) depolarized side scattering (DSS) properties of the hemozoin-containing red blood cells^17^,^18^. As an inherent advantage of flow cytometry, parasite counts can be determined in both cases directly. However, the number of GFP- and DSS-positive events may quantitatively differ as fluorescence detects all erythrocytic stages^13^, while DSS exposes only that fraction of parasitized RBCs that contain a sufficient amount of hemozoin^17^. Accordingly, the GFP positive events are used to assess parasitemia, while the DSS detection, similarly to MO and PCR yields indirect measure of the parasite burden.

The DSS and GFP values for series A, shown in Fig. 3, are scattered below the detection limit until 85–90 h pi, when both signals start to increase monotonically and the onset of the blood stage is confirmed with the first positive parasite counts of 0.04% and 0.12%, respectively. As expected the parasitemia counts determined by fluorescence were higher at any time point than the corresponding DSS values. In summary, both cytometric methods confirmed the onset of the blood-stage in the middle of the second erythrocytic cycle.

The flow cytometric curves of mice in series C show larger individual variation than those in series A (Fig. 4). The GFP and DSS signals exceed the detection limit between 95–120 h pi. In average the first positive GFP and DSS detections can be placed in the beginning and the second half of the 3rd cycle, respectively. The average parasitemia measured by fluorescence at the time points of the first positive detection is approx. 0.04% and the average DSS percentage is 0.07%.

The MO values of the A and B series (Fig. 1b) clearly exceed the detection limit at 66 h pi and 70 h pi, respectively. (Note that no measurements were performed on series B before 70 h pi). The MO measurements of the two series exhibit very similar tendencies and indicate that circulating parasites could be detected by the MO method as early as 66 h pi. The MO signals of the mosquito-infected mice in series C surpass the detection limit in the broad interval of 71–95 h pi. This large variation of the earliest detections is in agreement with the results of microscopy and flow cytometry, and is indeed expected due to the uncontrolled size of the inocula characterizing the natural infections. The delayed detection as compared to the A and B series suggests a lower initial parasite load as seen in the flow cytometric measurements. Nevertheless, the onset of the blood stage still could be detected in the first erythrocytic cycle for two out of three mice.

### Monitoring the progression of the blood-stage infection by the MO method

After establishing the first time points of erythrocytic parasite detection by microscopy, PCR, flow cytometry and the MO method, the progression of the infection at later time points was monitored by microscopy, flow cytometry and the MO method until day 6 and 7 pi in series A and C, respectively.

In series A and B the parasitemia values, as measured both by flow cytometry and microscopy, reached the level of 0.1–0.5% on day 4 pi (90–100 h timeframe) similarly to the results of Ploemen *et al.*^29^, where experiments were performed with similar initial sporozoite loads. In the case of series C this parasitemia range was reached only at the end of the fifth day (120 h) as a result of the considerably lower initial sporozoite load.

In Fig. 1b the MO signals of all three infected mice from series A show the same profile after exceeding the detection limit: the MO signal increases rapidly, interrupted by a distinct drop at 76 h. Following another steadily increasing period a drop of the increase rate can be identified between 90–109 h pi. The same behavior is observed for series B with only slight variations in the positions of the drops, observable at 80 h, 100 h and 125 h. While the drops at the early time points (76 h and 80 h) are clearly visible with a 65% and 75% reduction of the MO signal for series A and B, respectively, at later time points they are less pronounced.

In conclusion, the overall time dependence of the MO signal is very similar in all intravenously initiated infections. These results, supported by similar observations in flow cytometry and microscopy (Fig. 3a–c), suggest that the early blood stages of these controlled infections followed quite similar courses and that this progression could be reliably monitored by the MO measurements.

The MO curves of series C (Fig. 2b) show similar features to that of series A and B, but the signals of the individual animals reveal bigger variation as also found by the reference methods. It can be noted that the MO values of mice C-1 and C-2 exceed the detection limit already in the first erythrocytic cycle as found in series A and B, where mice were infected with considerably higher parasite loads.

The time evolution of the MO signal can be explained by considering that the total hemozoin concentration in the peripheral circulation is measured by this method. This includes crystals present within the parasites, free in circulation and/or inside phagocytic cells at the moment of blood sampling. Consequently, changes in the signal magnitude in the early phases of blood stage development are likely to result from two dynamic processes: i) the continuous production of hemozoin by the circulating parasites increases the MO signal and ii) the clearance of free hemozoin or hemozoin-containing phagocytes decreases the MO signal. If these two processes have comparable rates in a synchronous infection at a given parasite density, the MO signal is expected to pursue the following course: (i) gradual increase from the beginning of the first cycle, (ii) reaching a maximum at the end of the cycle when mature schizonts have maximal hemozoin content, (iii) decrease due to the rupture of iRBCs and the subsequent hemozoin clearance and (iv) turnover and increase due to the hemozoin production of the new generation of parasites.

In *P. berghei* infections the egress of merozoites from hepatocytes is expected around 48–52 h after sporozoite injection^6^,^7^ and the length of the asexual life cycle is 22–24 h^7^. Accordingly, the first intraerythrocytic cycle is expected to end between 72–76 h pi. This timeframe coincides well with the peak of the MO signal at approx. 72–75 h pi followed by the drop at 76–80 h pi observed both in series A and B. These observations indicate that the first parasites detected by the MO method around 66 h pi were approx. 14–16-hour-old parasites of the first synchronous life cycle. The additional drops observed with approx. 24-hour periodicity indicates that a partial synchronicity is preserved over a couple of cycles. The decreasing magnitude of subsequent drops indicates that synchronicity is eventually lost as known for *P. berghei* infections^7^,^30^.

In the first erythrocytic cycle the parasitemia levels were not quantifiable accurately by microscopy or by flow cytometry, but they can be estimated using the parasitemia values measured in the following cycles and the ~10-fold multiplication rate characterizing the first few erythrocytic cycles of the *P. berghei* ANKA parasites^31^,^32^. This multiplication rate is also confirmed by the 10-fold increase of the MO values between the ends of consecutive cycles. The average parasitemia for series A and B was in the range of 0.1–0.3% at the end of the second cycle. Accordingly, in these two series the parasitemia level of the first cycle was approx. 0.01–0.03%. For series C, the parasitemia values measured by GFP-detection at the end of the third intraerythrocytic cycle (Fig. 4b) were 0.6%, 0.2% and 0.04% for mouse C-1, C-2 and C-3, respectively. Accordingly, the average parasite loads for the three mice at the time of the first positive MO detection was approx. 0.004%.

### Monitoring parasite clearance by the MO method

In this study 5 mice (identified as T-1…5) with severe *P. berghei* infections were treated by the daily administration of chloroquine for seven days. Their MO signal was measured during the treatment and for nine consecutive days in order to study the clearance of the parasites and to determine the time interval after which the MO signal is reduced to the detection level.

**Figure 5:**
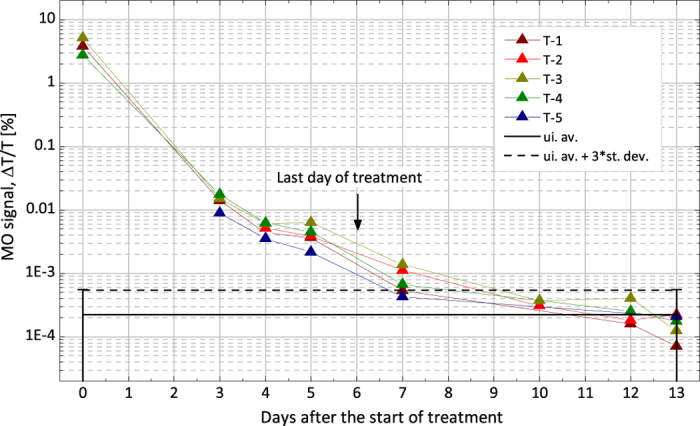
Monitoring parasite clearance during treatment by the MO method. The MO values of five mice were measured during and post treatment (T-series). Each colored triangle represents the MO value of a given mouse on a given day after the start of the treatment. The continuous black line represents the average of the MO values of five uninfected control mice measured in all time points. The dashed black line is the detection limit defined as the average plus three times the standard deviation of the uninfected values (for details see Methods section). The MO values of all treated mice reach the detection limit on day 10 and stay below it for the two consecutive measurements.

The first blood samples were collected from mice T-1, T-3 and T-4 on the first day of treatment (day 0 in Fig. 5). The corresponding parasitemia values, determined by microscopy, were 15%, 20% and 11% and the corresponding MO values were 3.76, 5.20 and 2.78, respectively. Since mice were anemic on the first day of treatment, no further blood collection was performed until the administration of the fourth dose of chloroquine (day 3). Subsequent blood samplings were performed as indicated in Fig. 5.

The MO signals of all the studied mice follow very similar decreasing trends. The substantial decrease in parasitemia (0.16% ± 0.07% and 0.06% ± 0.04% on day 3 and 4, respectively) caused by the administration of the first three doses of chloroquine is reflected in the rapid (approximately two orders of magnitude) decrease of the MO signal observed between day 0 and day 3.

On day 5, no viable parasites were observable by light microscopy in any of the mice. Consequently, the MO signal measured between days 5–7 is attributed to hemozoin crystals released from ruptured schizonts remaining in the circulation either freely or inside phagocytes^33^,^34^. The clearance rate with an approximate half-life of 24 hours is in agreement with the observations of mid- and long-term hemozoin kinetics reported for *in vivo P. berghei* infections^33^. By day 10 the MO signals in all treated mice was reduced to the detection limit and remained at this level for the consecutive two days, confirming the absence of the infection and yielding true negative diagnostic results.

## Discussion

The rotating-crystal magneto-optical diagnostic method has demonstrated excellent sensitivity to detect low concentrations of synthetic hemozoin crystals^27^ and low parasite densities of *P. falciparum in vitro* cultures^28^. Besides its *in vitro* sensitivity the method fulfills the most important technical requirements for in-field applicability: the measurement is automated, it is label- and reagent-free, and the device can be designed into a commercially available format^27^,^28^. In the present study the *in vivo* efficiency of the method was investigated by comparing its performance to well-known techniques.

In the investigated *P. berghei* infections the first erythrocytic parasites were already detectable at the late ring stage 66 h pi when mice were injected with 50,0000 sporozoites. In experiments when mice were subjected to mosquito bites the course of infection showed larger individual variation: in two cases the infection was detected at the end of the first cycle and in one case in the second half of the second cycle. Based on these results we conclude that the performance of the MO method is similar that of light microscopy. This performance is only exceeded by the laboratory-grade q-PCR (with detection as early as 56 h pi). In later time points the quantitative monitoring of the progression of the infection was also feasible with the MO method.

The blood stage of *P. berghei* infections has three important aspects that influence the hemozoin levels observable in the peripheral circulation: (i) the reticulocyte preference; (ii) the asynchronicity of later cycles and (iii) that the late schizonts tend to sequester in organs^35^. The first two properties are also typical features of the blood stage of *P. vivax* human infections. In this respect the current *P. berghei* experiments can be regarded as a simple model for the MO diagnostics of *P. vivax* infections.

The stage distribution of intraerythrocytic parasites in the case of *P. falciparum* infections, however, is different. In the latter case parasites older than mid-stage trophozoites tend to cytoadhere to the endothelium, thus the young forms present in the circulation contain little or no hemozoin. Clearly, this scenario is different from the *P. berghei* infection model, where only the very late schizonts sequester. However, the present study yielded two results with important implications for the detection of *P. falciparum* infections: (i) the first generation of parasites in the late-ring stage were already detectable and (ii) the MO results obtained during treatment of mice show that there is a substantial amount of hemozoin crystals circulating in the blood stream for a few days after schizont rupture. These observations indicate that the MO method has potential to detect human *P. falciparum* infections as well, either via the lesser amounts of hemozoin present in the blood sample inside the freely circulating late-stage rings or by detecting the hemozoin that is released from the sequestered schizonts after the rupture of iRBCs. During the treatment of infected mice we found that the period of false positive outcomes after successful treatments is limited to four days implying a similar scenario in the case of human infections.

Though the extrapolation of the results of rodent malaria experiments to human infections is never straightforward, we believe that the present study provides a solid basis for the implementation of in-field tests, which will assess the real performance of the method for the diagnostics of human infections.

## Methods

### Animals, parasites and treatment

In experiment series A and B, four and three BALB/c mice (Charles River, Spain) were infected, respectively, with the transgenic *P. berghei* ANKA (259cl2) that constitutively expresses GFP during the whole life cycle. Sporozoites were obtained by the disruption of the salivary glands of freshly dissected, infected female *Anopheles stephensi* mosquitoes and collected in DMEM (Dulbecco’s Modified Eagle Medium from GIBCO). Mosquitoes were bred at the insect facility of the Instituto de Medicina Molecular. Each mouse was inoculated by the retroorbital injection of 50,000 sporozoites.

Blood was collected from the tail vein according to the following schedule: in series A sampling started at the time point of 48 h pi and it was carried out in 5-hour intervals until 80 h pi; in series B sampling started only at 70 hours pi and two additional samples were taken at 75 h and 80 h pi. After 80 hours blood was collected daily in both series until the 5^th^ day post infection. Blood samples were collected for microscopy, PCR, flow cytometry and the MO method at each time point.

In series C three mice were subjected to five *P. berghei*-infected mosquitos for 20 minutes, individually. The method of blood sampling and its schedule was the same as in series A.

In the treatment measurements (T-series), five mice with severe *P. berghei* ANKA infection were treated by the daily administration of 7 mg/ml of chloroquine for seven days. Mice exhibited anemia on the first days of treatment and thus, no further blood was collected until the administration of the fourth dose of chloroquine (day 3). On this day blood samples from 4 mice were analyzed again by the MO method and by light microscopy. Further blood sampling and analysis was performed on days 4, 5, 7, 10, 12, and 13, as shown in Fig. 5.

### Ethics statement

This study was approved by the Ethical Committee of the Faculty of Medicine, University of Lisbon. All experiments involving animals were performed in compliance with the relevant laws and institutional guidelines. Animals were monitored daily and every effort was made to minimize their suffering. Upon completion of the experiments, mice were euthanized by administration of CO_2_ followed by cervical dislocation.

### Microscopic analysis

Blood parasitemia was monitored by the microscopic analysis of Giemsa-stained thin blood smears at each measured time point. Smears were fixed in absolute methanol and stained with 10% Giemsa-solution prepared in PBS 1X.

The presence of parasites in the early time points, i.e., before 90 h post-infection was determined by light microscopic examination of whole smears (approx. 20–40 fields, i.e. approx. 7,000–14,000 scanned RBCs) with 1000× magnification using a bright-field microscope (Leica, Solms, Germany) performed by two microscopists, independently. At later time points percentages of infected red blood cells and approximate age distribution of the parasites was assessed by light microscopic examination of 5–10 fields (approx. 1800–3600 RBCs).

### Magneto-optical measurements

All reagents were purchased from Sigma Aldrich (St Louis, Mo, USA) unless stated otherwise.

For the magneto-optical measurements 30 μl of blood was transferred from each mouse directly into 570 μl of lysis solution (0.066 V/V% Triton X-100 in 3 mM NaOH). The lysed sample was measured after 5 minutes, to enable the hemozoin crystals to be liberated from the RBC’s and from the parasites and become homogenously dispersed in the liquid^27^. MO measurements were performed using a volume of 450 μl from each lysed sample.

The scheme of the MO setup, as well as the underlying physical principles of the detection method, are described in detail in former studies^27^,^28^. Briefly, the lysed sample, filled into a cylindrical sample holder, is inserted into the center of a ring-shaped assembly of permanent magnets, which creates a strong uniform magnetic field (B = 1T) at the sample position. This magnetic field induces the co-alignment of the hemozoin crystals and when the magnetic ring is rotated, the co-aligned hemozoin crystals follow this rotation. During the measurement polarized light from a laser diode is transmitted through the sample in the direction perpendicular to the plane of the rotating magnetic field. The rotation of the co-aligned dichroic crystals gives rise to a periodic change in the transmitted intensity (ΔT), which – divided by the time-averaged intensity (T) – corresponds to the measured MO signal (ΔT/T in %). The overall time required for the full MO measurement process is a few minutes.

The level of the detection limit was determined as the mean plus three times the standard deviation of the MO signals measured on the following samples: one control mouse in series A, which was measured at the same time points as the infected ones of the same series; and four controls in series C also measured together with the infected animals of the same series. For clarity we only plot the average of the uninfected values (black line) and the corresponding detection limits (dashed black line) in graphs Figs 1b, 2b and 5. The individual results of the control measurements are plotted in Fig. 3 of the Supplementary information.

### DNA extraction and PCR analysis

At the selected time points 5 μl of blood was collected from the tail vein into 200 μl of PBS 1X. DNA extraction was performed using the DNeasy Blood & Tissue Kit (Quiagen, USA), according to the manufacturer’s instructions. Real-time PCR analysis was performed in duplicates using 2 μl of DNA and the iTaq Universal SYBR Green Supermix from Bio-Rad according to the manufacturer’s instructions. Expression levels of 18 s rRNA were normalized against the housekeeping gene seryl-tRNA synthetase (PbANKA_061540). Gene expression values were calculated based on the ΔΔ Ct method. Primer pairs used were: PbA 18S rRNA: 5′ GGAGATTGGTTTTGACGTT TATGTG3′ and 5′GGAGATTGGTTTTGACGTTTATGTG3′; PBANKA_061540: 5′ ATTGCTCAACCTTATCAAACTG3′ and 5′AGCCACATCTGAACAACCG3′.

### Flow cytometric measurements

A volume of 5 μl of blood was collected and diluted in 1 ml of PBS 1X at each time point for each mouse. This blood suspension was analyzed in the CyFlow® Blue instrument (Partec, Munster, Germany), which is equipped with a 488 nm excitation laser, and has detectors for forward scatter (FSC), side scatter (SSC), green fluorescence – FL1 (BP 535/35 nm), orange fluorescence – FL2 (BP 590/50 nm) and red fluorescence – FL3 (LP 630 nm). For this study the setup was modified as described previously^16^,^17^. Flow cytometry data were analyzed using FlowJo software (version 9.0.2, Tree Star Inc., Oregon, USA). Depolarizing events were defined in plots of side-scatter (SSC) versus depolarized-SSC as those with a signal above the background observed in the uninfected control. GFP positive cells were determined in green fluorescence (FL1) versus red fluorescence (FL3) plots.

In measurement series A one uninfected control was used to define GFP positive cells, which consisted of the ones with green fluorescence levels above the uninfected control. The detection limit of the whole DSS (GFP) measurement series (dashed lines in Fig. 3a,b) was determined as the average plus three times the standard deviation of the DSS (GFP) positive events measured on the uninfected control mouse in all of the investigated time points (not shown in the graphs).

In measurement series C the level of the detection limit (dashed lines in Fig. 4a,b) of the whole DSS (GFP) measurement series was determined as the mean plus three times the standard deviation of the DSS (GFP) positive events measured on the blood samples of four uninfected controls in the same time points as the infected mice (not shown in the graphs).

## Additional Information

**How to cite this article**: Orbán, Á *et al.* Efficient monitoring of the blood-stage infection in a malaria rodent model by the rotating-crystal magneto-optical method. *Sci. Rep.* **6**, 23218; doi: 10.1038/srep23218 (2016).

## Supplementary Material

Supplementary Information
