# Supplementary material for: Efficient monitoring of the blood-stage infection in a malaria rodent model by the rotating-crystal magneto-optical method
Source: Sci Rep. 2016 Mar 17;6:23218. doi: 10.1038/srep23218 (PMC4794716; doi:10.1038/srep23218)
Supplement: Supplementary Information [file srep23218-s1.pdf]

Supplementary information

Efficient monitoring of the blood-stage infection in a malaria rodent model by the rotating-crystal magneto-optical method

Ágnes Orbán, Maria Rebelo, Petra Molnár, Inês S. Albuquerque, Adam Butykai, István Kézsmárki

Table of contents

Figure S1 – Representative flow cytometric gating strategy in DSS measurements. 2

Figure S2 – Representative flow cytometric gating strategy in GFP measurements. 3

Figure S3 – The MO signals of five uninfected control mice. 4

Supplementary Figure 1.

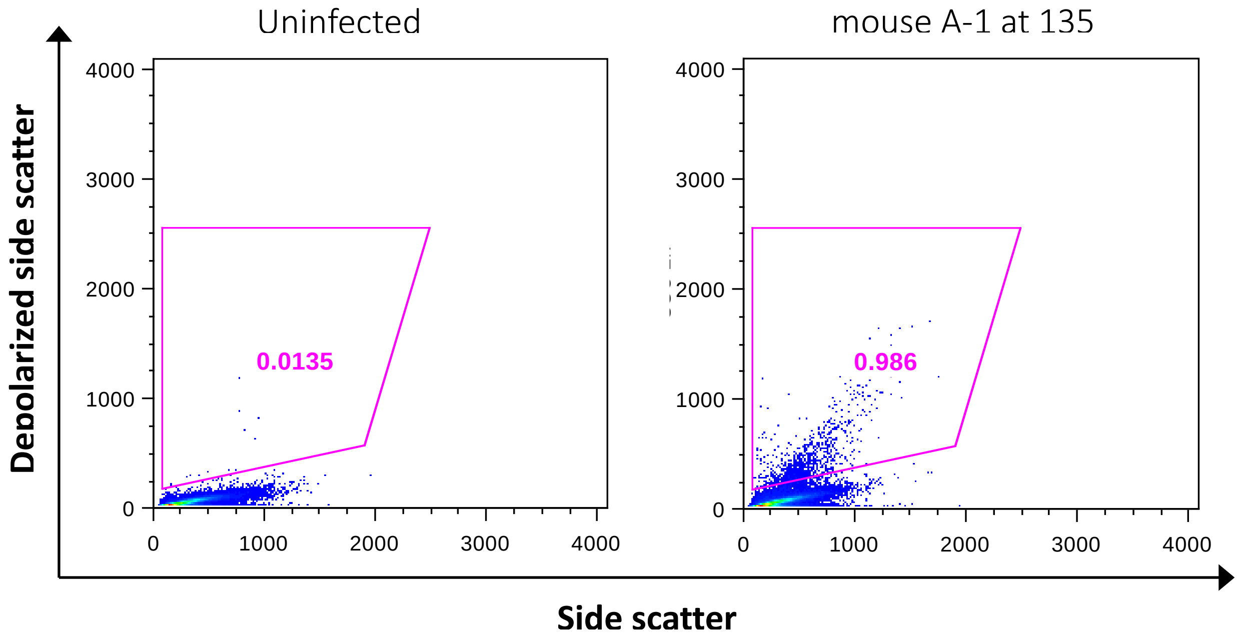

Figure S1 – Representative flow cytometric gating strategy in DSS measurements. Flow cytometric 2D plots of one uninfected and one infected sample from the infection experiment series 'A'. Depolarizing events were defined in plots of side-scatter (SSC) versus depolarized-SSC as those with a signal above the background observed in the uninfected control.

Supplementary Figure 2.

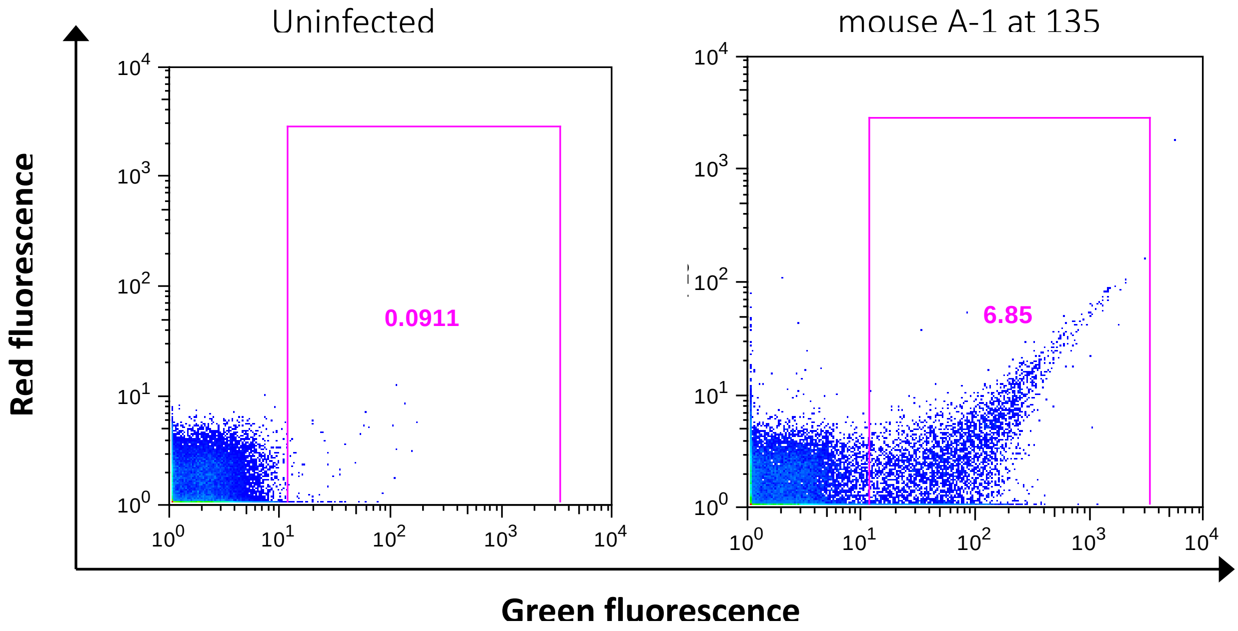

Figure S2 – Representative flow cytometric gating strategy in GFP measurements. Flow cytometric 2D plots of one uninfected and one infected sample from the infection experiment series ‚A'. GFP positive cells were determined in green fluorescence (FL1) versus red fluorescence (FL3) plots.

Supplementary Figure 3.

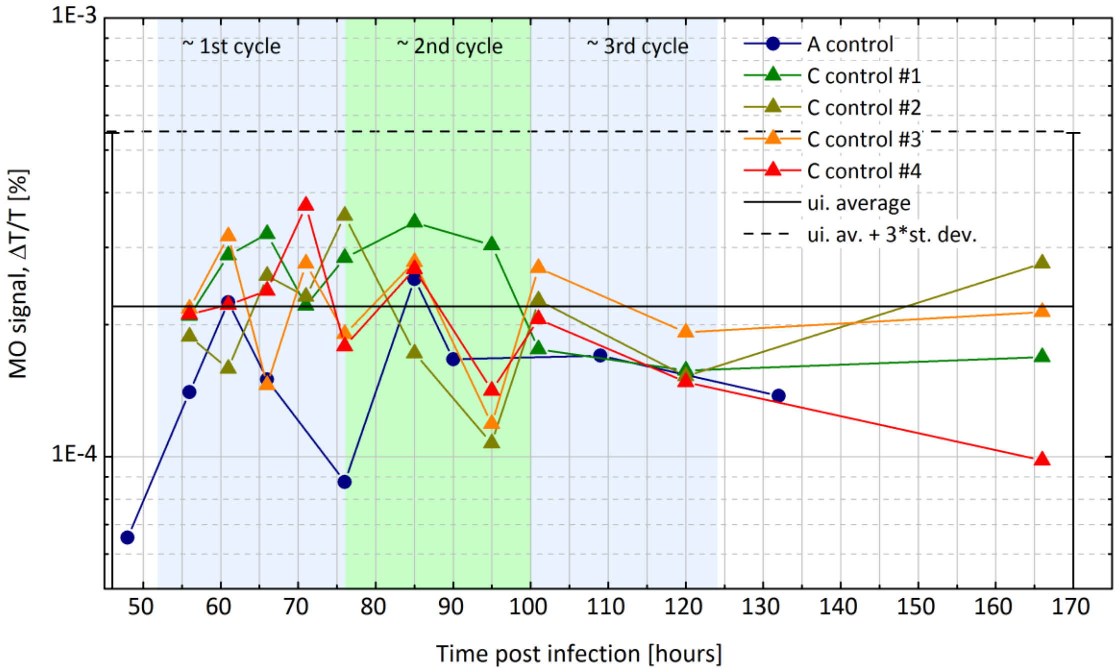

Figure S3 – The MO signals of five uninfected control mice. In series 'A' the MO values of one uninfected mouse (blue circle) were measured at the same time points as the infected ones. In series 'C' four controls (tringles) were kept and measured together with the infected mice. The solid black line is the average MO value of all these control measurements and the dashed black line is the 'average + 3*standard deviation' level, i.e. the detection threshold.
